# Supplementary figures and images for: Antagonistic relationship of NuA4 with the non-homologous end-joining machinery at DNA damage sites
Source: PLoS Genet. 2021 Sep 20;17(9):e1009816. doi: 10.1371/journal.pgen.1009816 (PMC8483352; doi:10.1371/journal.pgen.1009816)

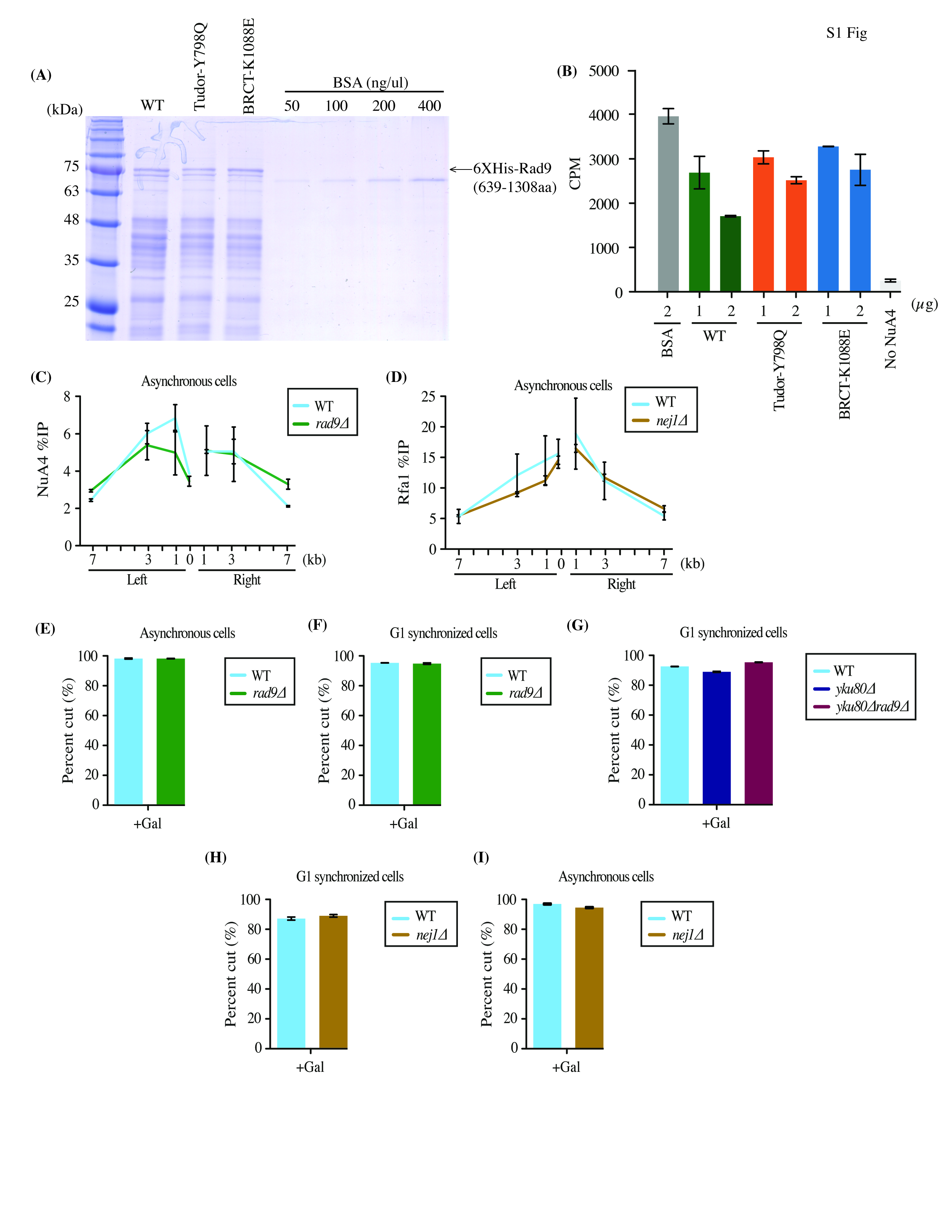

Supplement: S1 Fig — Related to Fig 1. (A) Purified recombinant Rad9 wild-type and mutants were resolved by SDS-PAGE and stained with Coomassie blue stain. Known concentrations of BSA were used to estimate the concentration of purified recombinant proteins using the ImageJ software. (B) Inhibition of NuA4-dependent acetylation of chromatin by Rad9 in vitro. The assays was performed as in Fig 1B but using more recombinant rad9 proteins. In these conditions the difference between WT and mutant Rad9 is more subtle because of mass effect, but still visible with 2ug. (C) ChIP-qPCR showing NuA4 enrichment around HO DSB at the MAT locus in wild type and rad9Δ in asynchronous cells. (D) ChIP-qPCR with antibody against Rfa1 showing no difference in resection at the MAT locus between wild type and nej1Δ in asynchronous cells. (E-I) HO cutting efficiency at the MAT locus after 3 hours of galactose induction in wild type and, rad9Δ in asynchronous cells (E), rad9Δ in G1 synchronized cells (F), yku80Δ and yku80Δrad9Δ in G1 synchronized cells (G), nej1Δ in G1 synchronized cells (H), nej1Δ in asynchronous cells (I). (TIF) [file pgen.1009816.s001.tif]

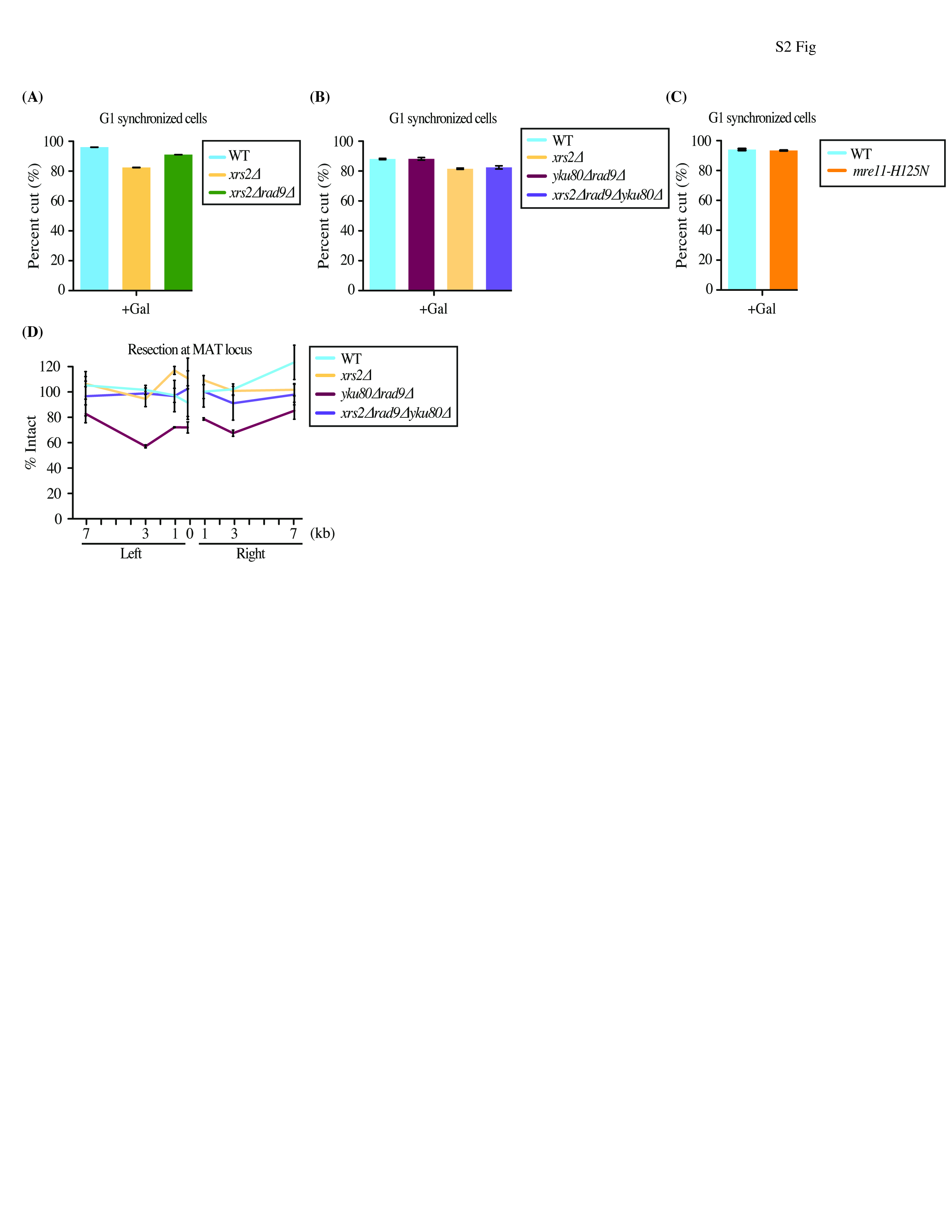

Supplement: S2 Fig — (A-C) HO cutting efficiency at MAT locus in G1 synchronized cells after 3 hours of galactose induction between wild type and, xrs2Δrad9Δ (A), xrs2Δrad9Δyku80Δ (B), mre11-H125N (C). (D) DNA end resection is directly measured by decreased DNA signal near the break in WT, xrs2Δ, yku80Δrad9Δ and xrs2Δrad9Δyku80Δ backgrounds. Hyper resection phenotype is clearly seen with yku80/rad9 double mutant but disappear in the absence of Xrs2. Resection is presented as % of Intact DNA determined by qPCR on genomic DNA at indicated sites and normalized to the negative control intergenic V. Cells were grown in YP-Raff till early log phase followed by addition of galactose for 3 hours to induce the DSB. (TIF) [file pgen.1009816.s002.tif]

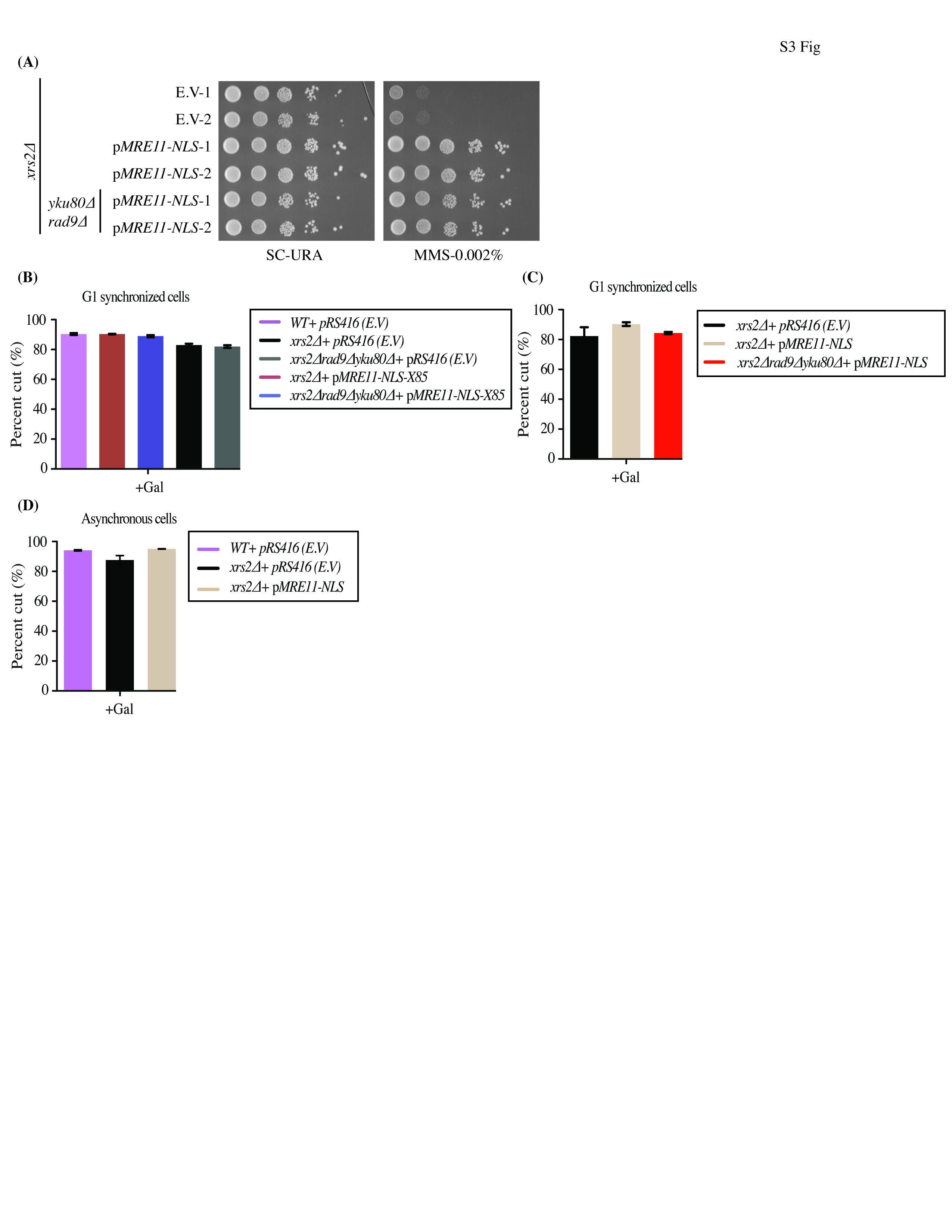

Supplement: S3 Fig — (A) Phenotypic analysis showing introduction of pMRE11-NLS suppresses the sensitivity of xrs2Δ cells to the DNA damaging drug MMS. 10-fold serial dilutions of log phase cells were spotted on synthetic complete -URA plates with and without MMS. (B-D) HO cutting efficiency at MAT locus after 3 hours of galactose induction in the indicated strains. (TIF) [file pgen.1009816.s003.tif]

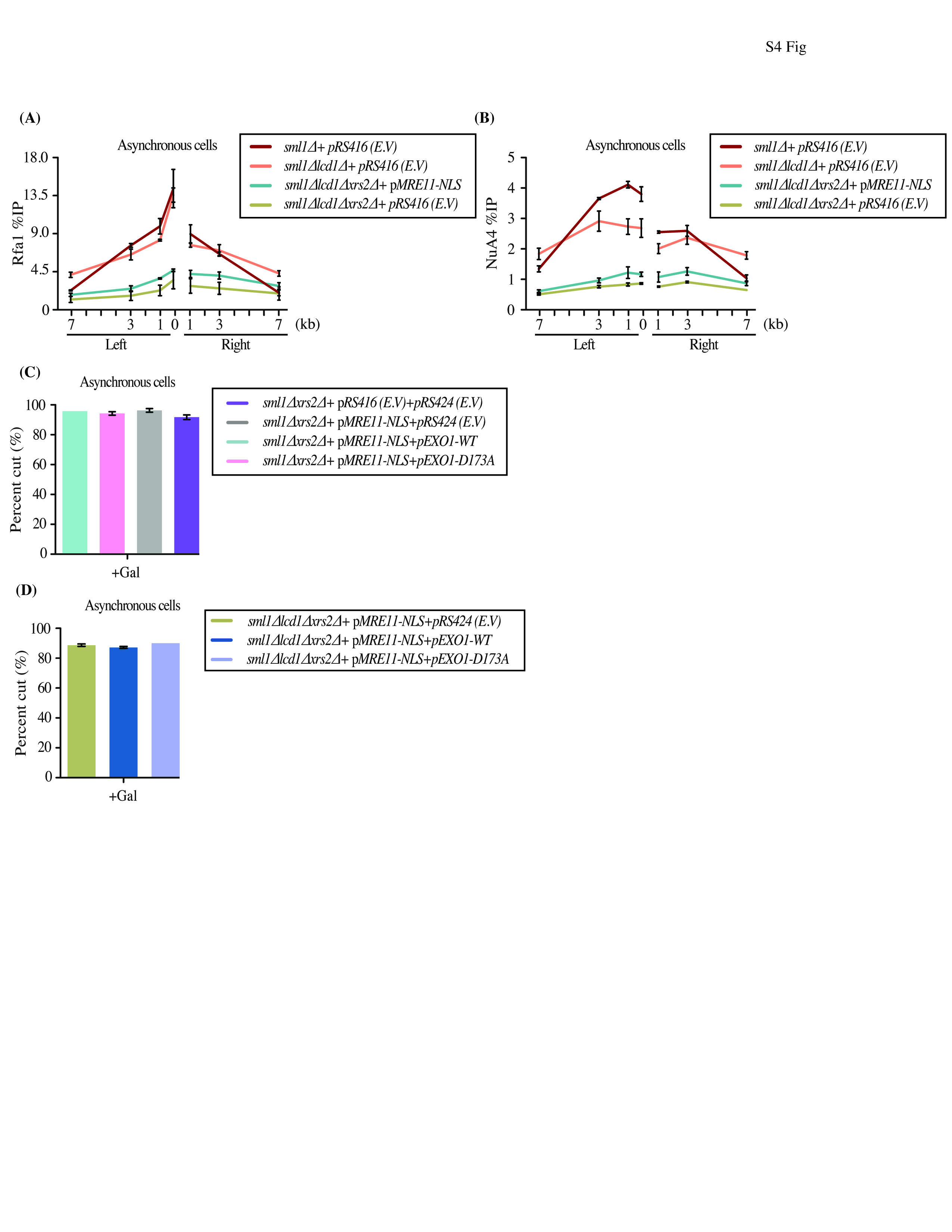

Supplement: S4 Fig — (A-B) ChIP-qPCR showing that the lcd1Δ xrs2Δ background results in loss of RPA signal (A), and NuA4 (B) at the HO induced DSB in asynchronous cells. The single mutant lcd1Δ (loss of Mec1) does not affect resection but shows slightly less NuA4 signal compared to wild type close to the break. (C-D) HO cutting efficiency at MAT locus after 3 hours of galactose induction in the indicated strains. (TIF) [file pgen.1009816.s004.tif]

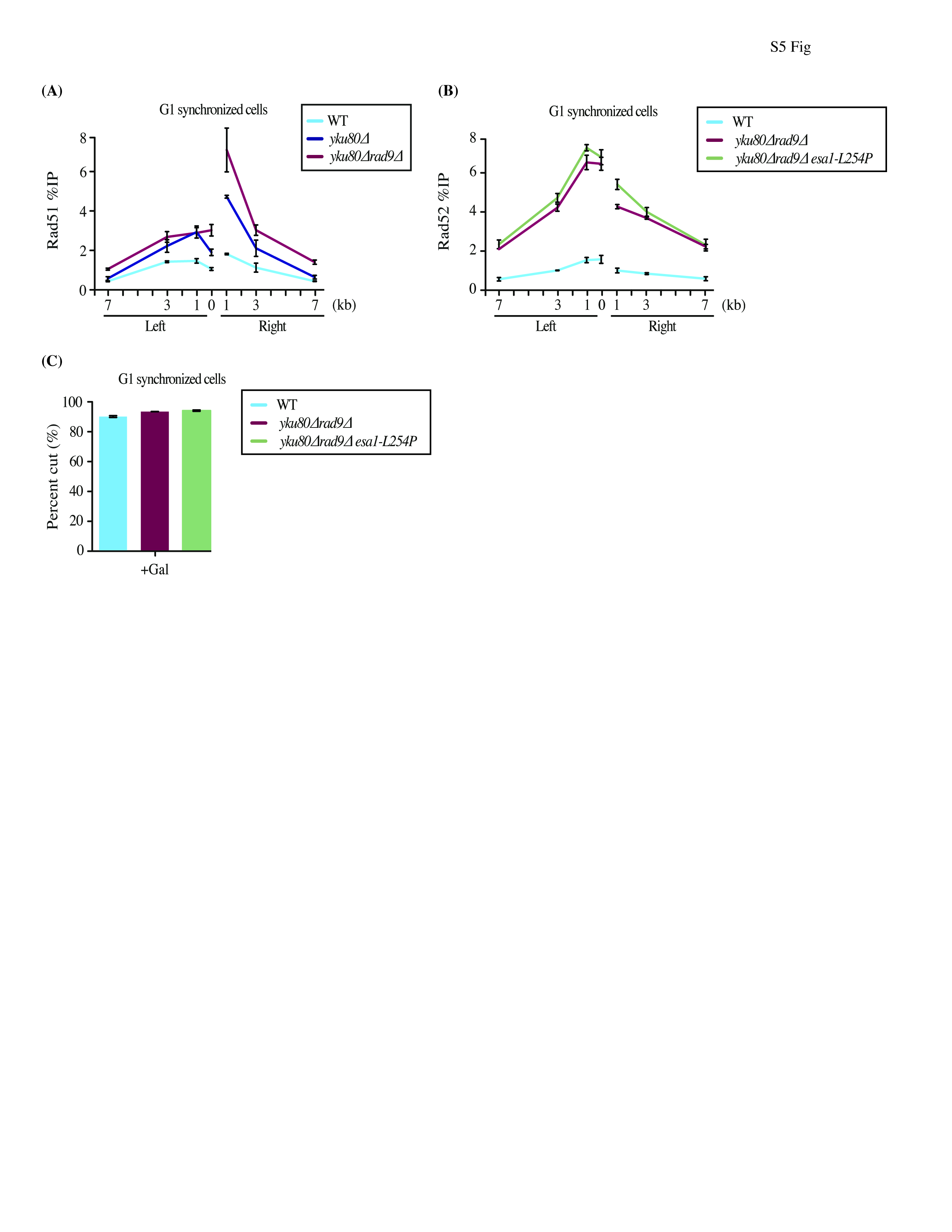

Supplement: S5 Fig — (A) ChIP-qPCR with a Rad51 antibody around the induced DSB at MAT locus in G1 synchronized cells. yku80Δ and yku80Δrad9Δ cells show higher signal compared to wild type, linked to hyper-resection. (B) ChIP-qPCR with a Rad52 antibody around the induced DSB at MAT locus in G1 synchronized cells. rad9Δyku80Δ and rad9Δyku80Δesa1-ts cells show higher signal for Rad52 than wild type but no significant difference by the addition of the esa1 ts allele. (C) HO cutting efficiency in wild type, rad9Δyku80Δ and rad9Δyku80Δesa1-ts cells at the MAT locus in G1 synchronized cells after 3 hours of galactose induction. (TIF) [file pgen.1009816.s005.tif]

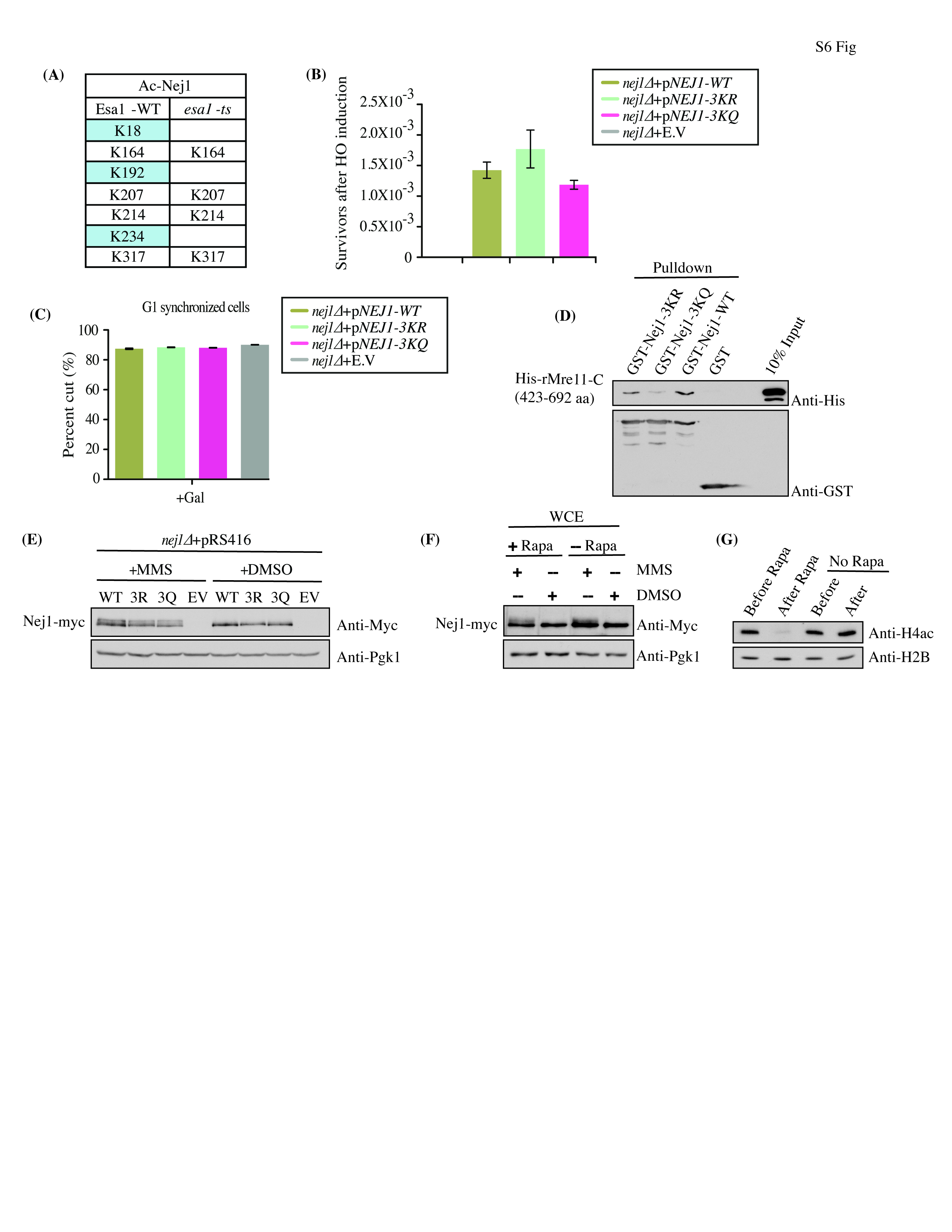

Supplement: S6 Fig — (A) Nej1 is acetylated in vivo on residues K18, K192 and K234 in a NuA4-dependent manner. Mass spectrometry analysis of Nej1 lysine acetylation sites in ESA1 wild type and temperature sensitive (ts) mutant. See full peptide sequences in S2 Table. (B) Nej1 wild-type and lysine mutants show similar ability to repair a chromosomal DSB induced at MAT locus by HO induction in the absence of HML/HMR donor sequence. (C) HO cutting efficiency at MAT locus in G1 synchronized cells after 3 hours of galactose induction in the indicated nej1Δ strains. (D) In vitro protein pull-down assay showing that recombinant wild type Nej1 and Nej1-3KR bind to the C-terminal region of rMre11, while this interaction is much reduced with Nej1-3KQ. Empty GST was used as negative control. (E) Nej1 lysine mutants do not affect the DNA damage induced phosphorylation of Nej1. Western blot analysis of whole cell extracts (WCE) prepared from indicated yeast strains treated with MMS (0.05%) or DMSO (control) for 2 hours. Pgk1 was used as loading control. (F) Western blot analysis on WCE showing depletion of Esa1 does not affect the DNA damage induced phosphorylation of Nej1. Esa1 was FRB tagged and depleted from the nucleus using the anchor-away system in presence of rapamycin [24]. This was followed by treatment of cells with MMS or DMSO. Pgk1 was used as loading control. (G) Western blot analysis of yeast WCE showing reduced acetylation of H4 in the anchor-away background with Esa1 FRB tagged after rapamycin treatment. H2B was used as loading control. (TIF) [file pgen.1009816.s006.tif]
