# Supplementary material for: Antagonistic relationship of NuA4 with the non-homologous end-joining machinery at DNA damage sites
Source: PLoS Genet. 2021 Sep 20;17(9):e1009816. doi: 10.1371/journal.pgen.1009816 (PMC8483352; doi:10.1371/journal.pgen.1009816)
Supplement: S1 Table — (DOCX) [file pgen.1009816.s007.docx]

**S1 Table: Yeast strains used in the current study**

| **Strain** | **Genotype** | **Reference** |
| --- | --- | --- |
| JKM139 | *MATa ho∆ hml∆::ADE1 hmr∆::ADE1 ade1-100 leu2-3;112 lys5 trp1::hisG ura3-52 ade3::GAL-HO* | [1] |
| QY2211 | JKM139 *bar1∆::NATMX* | This study |
| QY2212 | JKM139 *bar1∆::NATMX* *rad9∆::KANMX* | This study |
| QY2213 | JKM139 *bar1∆::NATMX yku80∆*::*TRP1* | This study |
| QY2214 | JKM139 *bar1∆::NATMX* *rad9∆::KANMX yku80∆*::*TRP1* | This study |
| QY2231 | JKM139 *bar1∆::LEU2* *rad9∆::KANMX yku80∆*::*TRP1 esa1-L254P::NATMX* | This study |
| QY2215 | JKM139 *bar1∆::NATMX nej1∆*::*TRP1* | This study |
| QY2216 | JKM139 *bar1∆::NATMX xrs2∆*::*TRP1* | This study |
| QY2217 | JKM139 *bar1∆::NATMX* *rad9∆::KANMX xrs2∆*::*TRP1* | This study |
| QY2218 | JKM139 *bar1∆::NATMX* *rad9∆::KANMX xrs2∆*::*TRP1 yku80∆*::*HPHMX* | This study |
| QY2219 | JKM139 *bar1∆::NATMX mre11-H125N::HPHMX* | This study |
| QY2220 | JKM139 *bar1∆::NATMX* + (pRS416) | This study |
| QY2221 | JKM139 *bar1∆::NATMX xrs2∆*::*TRP1* + (pRS416) | This study |
| QY2222 | JKM139 *bar1∆::NATMX xrs2∆*::*TRP1* + (pRS416 *MRE11-NLS*) | This study |
| QY2223 | JKM139 *bar1∆::NATMX* *rad9∆::KANMX xrs2∆*::*TRP1 yku80∆*::*HPHMX* + (pRS416 *MRE11-NLS*) | This study |
| QY2260 | JKM139 *bar1∆::NATMX xrs2∆*::*TRP1* + (pRS416 *MRE11-NLS-X85*) | This study |
| QY2261 | JKM139 *bar1∆::NATMX* *rad9∆::KANMX xrs2∆*::*TRP1 yku80∆*::*HPHMX* + (pRS416 *MRE11-NLS-X85*) | This study |
| QY1603 | JKM139 *sml1::NATMX* | [2] |
| QY2224 | QY1603 + (pRS416) | This study |
| QY2259 | QY1603 *xrs2∆::LEU2* + (pRS416) + (pRS424 *TRP1 2μ ori*) | This study |
| QY2258 | QY1603 *xrs2∆::LEU2* + (pRS416 *MRE11-NLS*) + (pRS424 *TRP1 2μ ori*) | This study |
| QY2256 | QY1603 *xrs2∆::LEU2* + (pRS416 *MRE11-NLS*) + (pRS424 *EXO1 TRP1 2μ ori*) | This study |
| QY2257 | QY1603 *xrs2∆::LEU2* + (pRS416 *MRE11-NLS*) + (pRS424 *EXO1-D173A TRP1 2μ ori*) | This study |
| QY1604 | JKM139 *sml1::NATMX lcd1::KANMX* | [2] |
| QY2225 | QY1604 + (pRS416) | This study |
| QY-2226 | QY1604 *xrs2∆::LEU2* + (pRS416) | This study |
| QY2227 | QY1604 *xrs2∆::LEU2* + (pRS416 *MRE11-NLS*) | This study |
| QY2228 | QY2227 + (pRS424 *TRP1 2μ ori*) | This study |
| QY2229 | QY2227 + (pRS424 *EXO1 TRP1 2μ ori*) | This study |
| QY2230 | QY2227 + (pRS424 *EXO1-D173A TRP1 2μ ori*) | This study |
| QY2600 | BY4741 *MATa his3∆1 leu2∆0 met15∆0 ura3∆0 nej1∆*::*KANMX* | This study |
| QY2232 | QY2600 + (pRS413) | This study |
| QY2233 | QY2600 + (pRS413 *NEJ1*) | This study |
| QY2234 | QY2600 + (pRS413 *NEJ1-K18R-K192R-K234R*) | This study |
| QY2235 | QY2600 + (pRS413 *NEJ1-K18Q-K192Q-K234Q*) | This study |
| QY2236 | QY2215 + (pRS416) | This study |
| QY2237 | QY2215 + (pRS416 *NEJ1*) | This study |
| QY2238 | QY2215 + (pRS416 *NEJ1-K18R-K192R-K234R*) | This study |
| QY2239 | QY2215 + (pRS416 *NEJ1-K18Q-K192Q-K234Q)* | This study |
| QY3572 | JKM139 *fpr1Δ::HphMX tor1-1::URA3 RPL13A-2*FKBP12::TRP1 FRB-ESA1::KanMX* | [3] |
| QY2240 | QY3572 *YKU80-13MYC::NATMX* | This study |
| QY2241 | QY3572 *NEJ1-13MYC::NATMX* | This study |
| QY2054 | BY4741 *MATa his3∆1 leu2∆0 met15∆0 ura3∆0* +pEGH(GAL-GST-Nej1) | Open Biosystem |
| QY2058 | BY4741 *MATa his3∆1 leu2∆0 met15∆0 ura3∆0 esa1∆::KanMX* +pSAPE5(HA-*esa1-L254P LEU2 ARS/CEN*) +pEGH(GAL-GST-Nej1) | This study |
| tNS2333 | hoΔ hmlΔ::ADE1 mat**a**Δ::hisG hmrΔ::ADE1 his4::[NAT-leu2(Asp718-SalI)] leu2::HOcs ade3::GAL::HO ade1 lys5 ura3-52 Ty*∆* | [4] |
| QY2266 | tNS2333 *rad51∆::KAN* | This study |
| QY2264 | tNS2333 *esa1-L254P::NATMX* | This study |
| QY2267 | tNS2333 *esa1-L254P::NATMX rad51∆::KAN* | This study |
| QY2265 | tNS2333 *rad52∆::NAT* | This study |

**References:**

1. Lee SE, Moore JK, Holmes A, Umezu K, Kolodner RD, Haber JE. Saccharomyces Ku70, Mre11/Rad50, and RPA Proteins Regulate Adaptation to G2/M Arrest after DNA Damage. Cell. 1998;94(3):399-409. doi: <https://doi.org/10.1016/S0092-8674(00)81482-8>.

2. Cheng X, Jobin-Robitaille O, Billon P, Buisson R, Niu H, Lacoste N, et al. Phospho-dependent recruitment of the yeast NuA4 acetyltransferase complex by MRX at DNA breaks regulates RPA dynamics during resection. Proceedings of the National Academy of Sciences. 2018;115(40):10028-33. doi: 10.1073/pnas.1806513115.

3. Kollenstart L, de Groot AJL, Janssen GMC, Cheng X, Vreeken K, Martino F, et al. Gcn5 and Esa1 function as histone crotonyltransferases to regulate crotonylation-dependent transcription. Journal of Biological Chemistry. 2019;294(52):20122-34. doi: 10.1074/jbc.RA119.010302.

4. Jain S, Sugawara N, Haber JE. Role of Double-Strand Break End-Tethering during Gene Conversion in Saccharomyces cerevisiae. PLoS Genet. 2016;12(4):e1005976. Epub 2016/04/14. doi: 10.1371/journal.pgen.1005976. PubMed PMID: 27074148; PubMed Central PMCID: PMC4830573.
