# Supplementary material for: Antagonistic relationship of NuA4 with the non-homologous end-joining machinery at DNA damage sites
Source: PLoS Genet. 2021 Sep 20;17(9):e1009816. doi: 10.1371/journal.pgen.1009816 (PMC8483352; doi:10.1371/journal.pgen.1009816)
Supplement: S2 Table — List of Nej1 peptides containing an acetylated lysine residue confidently identified by mass spectrometry after affinity purification from extracts of WT and esa1 ts cells and trypsin digestion. Total peptide sequence coverage obtained were 77 and 83% respectively. (DOCX) [file pgen.1009816.s008.docx]

**S2 Table: Acetylated peptides detected by mass spectrometry in *ESA1* and *esa1* ts strains**

| **Yeast strain** | **Sequence position** | **Peptide sequence** |
| --- | --- | --- |
| *ESA1*-WT | aa 7-19 | GQQLSDAEWCVKacK |
|  | aa 163-174 | QKacCLDFLLISLR |
|  | aa 182-208 | VISQWAPENSKacNYESLQQCTDDDIIKK |
|  | aa 193-208 | NYESLQQCTDDDIIKacK |
|  | aa 213-226 | GKacFQHQEFLADSLK |
|  | aa 233-240 | NKacFQDVSR |
|  | aa 305-319 | LENFSESEATPEKacTK |
| *esa1-L254P* | aa 163-174 | QKacCLDFLLISLR |
|  | aa 193-208 | NYESLQQCTDDDIIKacK |
|  | aa 213-226 | GKacFQHQEFLADSLK |
|  | aa 305-319 | LENFSESEATPEKacTK |
