# Supplementary material for: Antagonistic relationship of NuA4 with the non-homologous end-joining machinery at DNA damage sites
Source: PLoS Genet. 2021 Sep 20;17(9):e1009816. doi: 10.1371/journal.pgen.1009816 (PMC8483352; doi:10.1371/journal.pgen.1009816)
Supplement: Attachment — Submitted filename: ResponsetoReviewers.pdf [file pgen.1009816.s010.pdf]

Response to reviewers' comments:

Reviewer #1:

In this paper, Ahmad et al. study DSB processing and NuA4 binding in the G1 phase. They use rad9 and ku mutants to achieve conditions of decreased end protection and increased resection and show that increased resection goes hand-in-hand with increased NuA4 binding. Interestingly, they show that an esa1 acetylation defective mutant reduces the hyper-resection of a rad9 (or rad9 ku80) mutant and conclude that NuA4 acetyltransferase activity is important to stimulate DNA end resection in G1. Presumably this same role would operate in S/G2 or other situations where resection occurs at a DSB, but this wasn't addressed. They characterize the resection requirements thoroughly, and establish that recruitment of NuA4 depends on Xrs2, but ultimately this may be due to an indirect effect of lack of Mre11 entry to the nucleus and resection in the Xrs2 mutant, and maybe Ddc2 is the actual NuA4 interactor. The Ddc2 involvement data is indirect and the role of Xrs2 vs Ddc2 is not clearly distinguished, either experimentally or in the writing. Finally, they establish that end-joining factors Nej1 and Yku80 are NuA4 (Esa1) targets, and acetylation of Nej1 reduces its interaction with other NHEJ factors. This could provide a way to control DSB repair pathway choice. This part of the paper was under-developed and not connected mechanistically to the rest.

Overall, this paper describes 2 somewhat separate roles for NuA4 in NHEJ, neither of which was fully characterized. (1) (Fig. 5) direct acetylation of members of the Ku end-joining complex to regulate their interactions (and possibly stability on the DNA, though this was not directly shown). (2) Figs 1-4. Role of NuA4 in resection. This is presumably not a normal G1 role but can occur in G1 in abnormal circumstances such as when end protection is gone (ku80 missing) or resection is uncontrolled (rad9 deletion). However, it was not clearly established how NuA4 is recruited (indirect evidence that it could be through Ddc2 interaction) or what it is acetylating to increase resection (what are the targets). Since this was done in conditions where the end was already unprotected, presumably the targets are RPA or histones or some other internally binding protein. Thus, this part of the story seems un-connected to the role of acetylating the Ku/end-joining factors. The authors could consider separating these data into two manuscripts and more thoroughly characterizing each pathway.

We thank the reviewer for his comments since they pointed us to change the text to more clearly state what are the key goals and findings of our study. Indeed, it seems that the reviewer misunderstood the major goal of our study, which was the functional relationship between the NHEJ machinery and the NuA4 complex for the repair of DNA DSBs.

-First, we were not trying to demonstrate NuA4 role in overall DNA end resection at double-strand breaks. This was proven as part of our other study just published and showing that, in asynchronous cells, the combined recruitment and action of NuA4 with another major HAT complex, SAGA, are essential for DNA end resection and recombination to occur (Cheng *et al*. PLoS Genetics 2021). The goal here was to understand the regulatory cross-talk between NuA4 and the NHEJ machinery, suggested by previously published genetic interactions and work in mammalian cells. Yeast cells blocked in G1 were ideal to investigate the regulatory cross-talk as NHEJ is the main repair pathway used to repair DNA breaks in this cell cycle phase and yeast is a powerful system for genetic manipulation. While NuA4 is normally recruited at DNA breaks in

S/G2, we now show that it can also happen in G1 when the NHEJ machinery is disrupted, supporting the hypothesis of an antagonistic relationship.

-Second, NuA4 recruitment in G1 when the NHEJ machinery is disrupted is linked to the now allowed DNA end resection. In our previous work (Cheng *et al.* PNAS 2018), we had shown through *in vitro* and *in vivo* experiments that NuA4 initial recruitment in S/G2 was likely mediated by a phospho-dependent interaction with the Xrs2 subunit of the MRX complex, followed by spreading on each side of the break during resection. Thus, we investigated if NuA4 recruitment in G1 was following the same mechanism. While the dependency on Xrs2 was again confirmed, a more thorough analysis with genetic suppressors of *XRS2* deletion uncovered an additional mechanism of recruitment likely linked to the spreading of NuA4 during end resection. We had previously shown that the ATM-related subunit of NuA4, Tra1, can physically interact *in vitro* with both Xrs2 and Ddc2/Lcd1 (PNAS 2018). On the other hand, deletion of *DDC2/LCD1* had no apparent effect on NuA4 recruitment in S/G2. Now, we clearly show (supported by additional data, **new Fig. 3A-B**) that Ddc2/Lcd1 plays a role in NuA4 recruitment in G1 when Xrs2 requirement is genetically bypassed.

In parallel, as we have shown that NuA4 plays a role in DNA end resection in S/G2 through acetylation of chromatin, RPA and maybe additional factors (PNAS 2018, PLoS Genetics 2021), we now show that it also affects hyper-resection seen in G1 when the NHEJ machinery is disrupted. That directed us to speculate a role of NuA4 in another resection-dependent repair pathway, single-strand annealing (SSA), that could occur in G1 since a sister chromatid is not available for homologous recombination. Indeed, we have now included an assay that demonstrates a role of NuA4 in SSA when regions of homology are separated by 20kb in cis **(new Fig. 5F**).

-Third, in parallel to our data showing that the NHEJ machinery blocks NuA4 recruitment to DNA breaks in G1, we investigated if NuA4 can inhibit the NHEJ pathway and prove a truly antagonistic relationship. A first hint was published a few years ago in a large-scale study showing that purified NuA4 could acetylate recombinant Nej1 on a microarray (Lin et al. Cell 2008). We now show data in the revised manuscript that confirm acetylation of Nej1 by NuA4 *in vitro* and acetylation of Nej1 in vivo (**new Fig. 6A-B, new S2 Table**). Our results clearly demonstrate that NuA4 can indeed inhibits NHEJ at least in part through acetylation of Nej1, and probably Ku80, most likely disrupting the assembly of the machinery at the DNA break. Overall, our results confirm our hypothesis of a strong antagonistic relationship between the NHEJ machinery and the NuA4 complex, each one capable of inhibiting the other to favor end-joining versus resection-based repair of DNA double-strand breaks.

As an added note, during revisions, we tried to map the NuA4-dependent acetylation sites on Ku80 *in vivo*. While a few acetylated peptides were tentatively identified by mass spectrometry, none passed the confidence threshold required to create mutants. Further work will be necessary to investigate if Ku80 acetylation by NuA4 can also inhibits NHEJ, like Nej1 acetylation. Importantly, Ku70 acetylation in higher eukaryotes has been suggested to inhibit its function (doi: https://doi.org/10.1101/403485). We did check Ku80 signal at the HO break by ChIP-qPCR in the presence or the absence of NuA4 and could not see a significant change of signal (see results included below). This suggests that Ku80 acetylation does not affect its binding the DNA ends, most likely affecting its interaction with the rest of the NHEJ machinery, and/or that the proportion of Ku80 that is acetylated is too low to change the signal of total Ku80 detected at

the DSB. Unfortunately, we could not monitor Nej1 or Lif1 presence at the HO break since ChIP-qPCR with different tags did not provide in our hands a specific signal over background. If required, we can include the Ku80 ChIP data as supplemental information. We did not at this time since we feel it may confuse the reader.

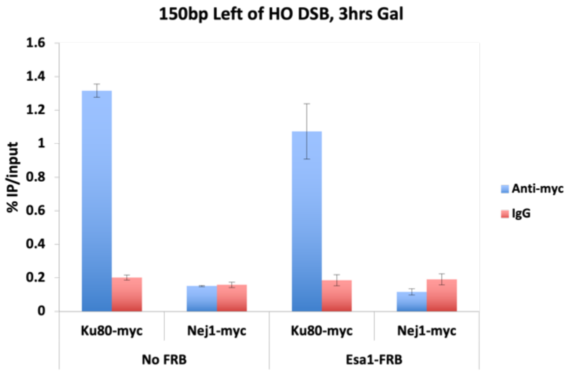

Fig. 1B. Is the difference between wt and mutant rad9 conditions statistically significant?

This *in vitro* experiment is used to introduce the questions that are asked in the study. It follows the previously published genetic data that linked NuA4 and Rad9 (Clarke *et al.* MCB 1998, Javaheri *et al*. PNAS 2006), in parallel to our work in mammalian cells on the competitive binding of TIP60 and 53BP1 to chromatin for DSB repair pathway selection (Jacquet *et al.* Mol Cell 2016). It shows for the first time that binding of Rad9 to native chromatin can inhibit NuA4-dependent acetylation *in vitro*. As binding of Rad9 to chromatin near DSBs requires H2A-S129ph and H3K79me3, we also used Rad9 proteins mutated for the domains responsible for reading these marks, to validate the binding. We agree that the statistically significant inhibition of NuA4 activity seen with 1ug of Rad9 does not seem to be much reduced by the single mutants. On the other hand, with 2ug of Rad9, the mutants have significantly less inhibitory effect on NuA4. Nevertheless, we repeated the experiment using less Rad9 proteins, closer to a 1:1 ratio with chromatin in the assay (0.25-0.5ug of Rad9 vs 0.5ug of chromatin from MMS-treated cells). In this situation it is even clearer that Rad9 dependent inhibition of NuA4 activity is significantly decreased/lost with the mutants. This result is now included as **new Fig. 1B** while the old assay with more Rad9 is moved to supplemental S1B Fig.

Fig. 2E. The Mre11-H125N data shows that NuA4 is still recruited when resection is less robust (resection is not abolished in this mutant). However, I don't see how this shows that Xrs2 is the protein recruiting NuA4. And the data in Fig. 3 indicate that the main role of Xrs2 re. NuA4 recruitment is to bring Mre11 into the nucleus to start the resection process. Therefore, the conclusion of an Xrs2 direct role is not substantiated here. However, previous data showed a direct interaction between Xrs2 and NuA4 subunit Tra1 and concluded there was direct recruitment by Xrs2. This discrepancy is not directly addressed in the text, leading the reader confused about what the authors believe is the role for Xrs2.

Based on the reviewer's comments, we could see that better explanations were required, and we changed to text accordingly. In our earlier work (PNAS 2018), we concluded that NuA4 recruitment occurs in 2 steps at DSBs in S/G2, first through a physical phospho-dependent

interaction with Xrs2 (MRX) at the break, and second alongside DNA end-resection. With the new results obtained here, we propose that the initial phase of NuA4 recruitment is still dependent on Xrs2 and not resection (as shown with Mre11-H125N in Fig. 2), whereas, once resection occurs, NuA4 can also be recruited via an Xrs2-independent manner, most likely through a physical similar interaction with Ddc2/Lcd1 (as shown in Figs 3-4).

Fig. 3 text. I think the authors are implicating Lcd1/Ddc2 as the actual NuA4 recruiter, but it is not clearly stated how the absence of Mre11/Xrs2 would lead to insufficient Ddc2 recruitment. I assume it is indirect, that you need Mre11-dependent resection and RPA loading to recruit Ddc2, which in turn recruits NuA4. This could be more clearly written in the text. (side note: isn't Ddc2 the more commonly used name?)

As for the previous comment, we are not arguing that Ddc2/Lcd1 is the actual NuA4 recruiter. Based on our data we are arguing that it is both Xrs2 and Lcd1/Ddc2, linking it to the 2-steps recruitment of NuA4 we initially described (PNAS 2018). Xrs2/MRX is the initial recruiter while Lcd1/Ddc2 can be involved during DNA end resection. Again, the text has been changed to explain things more clearly.

Fig. 3E-F. <u>A NuA4 and RPA ChIP in the lcd12sml1 double should be done to show that Ddc2/Lcd1 is responsible for NuA4 recruitment. If there isn't enough resection in G1 cells, then ku80 and/or rad9 can be also deleted.</u> This seems much more straightforward than the experiment done, which doesn't show very clear results. The "increased resection" seen when adding in the pEXO1 plasmid is very subtle. And <u>the experiment in 3F doesn't have a Lcd1+ condition to compare to, which needs to be included.</u> Altogether, the data supporting recruitment of NuA4 by Lcd1/Ddc2 is quite indirect. Since this is a major point of the paper, this conclusion should be better supported. Confusingly, when I looked back at the data from the previous paper (ref 23), it turns out it was already shown that deletion of LCD1 had no effect on NuA4 recruitment to an HO break using a similar ChIP method. So why are the authors now speculating that Lcd1 binding is a second pathway for NuA4 recruitment to breaks?

As discussed above, Lcd1/Ddc2-dependent recruitment of NuA4 is only observed when Xrs2 is bypassed. That is why we did not observe it previously in asynchronous cells (PNAS 2018), nor would we observe it if we delete only *LCD1/DDC2* with *YKU80/RAD9* in G1 cells. It may have to do something with how the MRX complex is spreading along the DNA with RPA (Seeber et al. Mol Cell 2016; Gnugge and Symington Genes Dev 2017). We did perform the requested experiment with the Lcd1+ condition to compare and show that NuA4 recruitment in bypassed Xrs2/increased resection conditions is dependent on Lcd1/Ddc2 (**new Fig. 4A-B, compared to 4C-D**). So yes, we are suggesting that Lcd1/Ddc2 binding is a second pathway for NuA4 recruitment at DSBs.

To further support that an in vivo role of Ddc2 is to recruit NuA4, it would be helpful to delete an interaction domain on one of the proteins. For example, mutation of the Tra1 PIKK domain or the interacting Ddc2 domain to show that NuA4 recruitment to a DSB in is abolished. This could also get around any potential poor growth issues in using lcd1sml1rad9 triple mutants in the above suggestion (though lcd1sml1ku80 should not be sick).

Tra1 is essential for viability, and it has been shown that mutations throughout its PIKK/FATC/FRB/FAT domains are lethal as well (Knutson and Hahn MCB 2011). Thus, dissecting the exact domains of interaction between Lcd1 and Tra1/NuA4 for recruitment of the complex when Xrs2 is bypassed would need a large amount of additional work that seems outside the scope of this study. It will likely be more adequate for future studies in parallel with dissecting the Xrs2-Tra1/NuA4 interaction.

It would be very interesting to combine the Xrs2-phospho interaction site implicated previously (ref 23) in NuA4 recruitment with a Ddc2 mutant to see if the two recruitment pathways work together or independently. Or does the Xrs2 phosphorylation allow Ddc2 recruitment, and so the former finding was also indirectly related to Ddc2 recruitment? Does NuA4 acetylation of RPA need to be included in the model?

We are not sure to fully understand these suggestions. While we have mapped multiple DNA damage-induced phosphosites on NuA4 subunits, we still have not found which one or which combination of sites is required for the interaction with Xrs2 in vitro (PNAS 2018). While Xrs2 FHA domain does interact with NuA4, full binding also requires the BRCT domain. For Lcd1/Ddc2, we do not know which part of the protein is involved as it does not contain such domains. Furthermore, mutating the FHA domain of Xrs2 will not help much as it has other functions in the repair process. It would indeed be interesting to see if NuA4-dependent acetylation of RPA plays a role for Lcd1/Ddc2 function during resection. While the questions are very interesting, we again feel that addressing them will require a large amount of work, while the current study is in fact focused on the cross-talk between NuA4 and the NHEJ machinery.

Even if the role of Xrs2 vs Ddc2 in NuA4 recruitment is not addressed experimentally, it should be more clearly addressed in the writing. The introduction says that Xrs2 was previously implicated in NuA4 recruitment, and this was the premise of the experiment. But in the end, it seems that effect may be indirect (e.g no Xrs2, no Mre11 import to nucleus, no resection, so no RPA and Ddc2 loading to recruit NuA4). Altogether, the role of Xrs2/Mre11 versus Ddc2 needs to be clarified, taking into account both previously published and current data.

We certainly agree with the reviewer asking for important clarification in the text since his attention has been focused on NuA4 recruitment and resection. We have now changed the text to clarify the conceptual impact of the data in the current study on our previous model presented in PNAS 2018. Again, we are not arguing that Lcd1/Ddc2 is the recruiter of NuA4 while Xrs2 is not. We see dependency on Lcd1/Ddc2 only in specific conditions.

Fig. 5B is an experiment to show that Nej1 acetylation is important for NHEJ. The plasmid data is clear, but it would be important to check the phenotype in a chromosomal NHEJ assay. Similarly, it would be important to show a functional consequence on NHEJ upon the Esa1 depletion used in Fig. 5G that results in decreased Ku80 acetylation. These data are important, since a main point of the paper is that NuA4 acetylation of NHEJ factors regulates NHEJ. The title of this section "Acetylation of Nej1 by NuA4 in the regulation of DSB repair pathway choice" is misleading though, since a regulation of pathway choice is not shown.

These are nice suggestions, but the proposed experiments are not so easy as to obtain clear results. We did try a chromosomal NHEJ assay using growth assays (see S6B Fig) and/or qPCR

to detect repair by end joining. But the small % of uncut HO site varying between conditions (+/- NuA4) makes it very difficult to be fully confident about increased NHEJ. As we mentioned in the manuscript, the colonies that survive are either from the cells that did not cut the HO site or the ones doing alt-NHEJ but not classical NHEJ. In another study we tested the effect of depleting a non-essential subunit of NuA4 on the plasmid-based NHEJ assay and saw no effect (Fig S2A, Cheng et al. PLoS Genetics 2021). But this assay requires growth on plates which is not possible with essential subunits like Esa1 used here.
For the role of Ku80 acetylation, as discussed above, we were not able at this time to precisely mapped confident acetylation sites by mass spectrometry. We did look at the effect of Esa1/NuA4 depletion on the presence of Ku80 at the HO break but could not see a significant effect (data can be included if judged necessary). Again, this is likely due to a relatively small portion of Ku80 being acetylated to start with in these conditions, and/or a role of acetylation not to block Ku80 binding but instead the assembly of the NHEJ machinery.
We did not feel that the title of the section on Nej1 was misleading since we show, along with NHEJ plasmid relegation assay, both *in vivo* and *in vitro* how the Nej1 acetyl mutants impact resection and binding with core NHEJ factors. New included data also now show that NuA4 can play a role in SSA, a resection-based repair pathway that can occurs in G1 when the NHEJ machinery is disrupted (**new Fig. 5F**). Nevertheless, we changed the title to tone it down.

Reviewer #2:

Ahmad et al., describes the antagonistic relationship between the Non-Homologous End-Joining machinery at an HO-induced DSB. The authors also identify Ku and Nej1 as NuA4 targets and through mutagenesis they show data supporting the model that acetylation helps remove NHEJ factors as a mechanism to promote HR. The experiments are high quality and the work contributes to the DSB field. A few controls are requested and further explanation is needed in some areas.

We are of course delighted that the reviewer found our data of high quality and an important contribution to the DSB field. We have added the controls and further explanation as requested.

- *The interpretation of data with xrs2Δ needs revision as the MRX complex does not assemble at breaks and other factors binding to MRX would not be recruited either. Line 160 (p.8) '... due to the loss of interaction with Xrs2, or the loss of resection itself.' is a generalization and does not capture the complexity as mre11Δ would give the same result. Comparing xrs2Δ to mre11-H125N indicates that the nuclease activity is not required for NuA4 recruitment. As the authors show NuA4 interacts with Xrs2-N-term (PNAS) –incorporation of pMRE11-NLS-X85 or X164 into their work would clarify a number of questions.*

    Mre11 is not recovered at the DSB in xrs2Δ and it is largely reduced in pMRE11-NLS, but restored when fragments of Xrs2, X85 or X164, are fused to Mre11. Presumably these would not interact with NuA4. For Fig. 3, include pMRE11-NLS-X85 or X164. This would clarify the interpretation of data in Fig. 2 and 3.

    We are very grateful to the reviewer for these very important insights and great suggestion for a new experiment to clarify the findings/interpretation. We have

now included new data in **Fig. 3C-D/S3B Fig** using pMRE11-NLS-X85 (from Oh et al. Cell Rep 2018), that clearly and more definitely support our previous conclusions, i.e. that NuA4 can be recruited in the absence of an interaction with Xrs2.

For Fig. 3A, does the IP WT + pRS416 (EV) in G1 give the same enrichment as WT in Fig. 2? Please include this as well *xrs2Δ rad9Δ ku80Δ* +pRS416 (EV). This will help with the interpretation of data between Fig. 3A and Fig.2C.

These controls have now been included in **new Fig. 2C-D and 3C-D**.

Does xrs2Δ have the same additive effect with rad9Δ that yku80Δ has for NuA4 Rfa1 and Rad52 localization (Fig. 1G-H and 4B)?

We suppose the reviewer is asking about *nej1Δ* instead of *xrs2Δ* based on the panels mentioned, to check if combining *nej1* with *yku80* or *rad9* gives similar results (increased NuA4, Rfa1, Rad52) as combining *rad9* with *yku80.* We think it should have similar increased effects. However, it might be less than what we see in *rad9 yku80* double deletion. Single *nej1* deletion gives less NuA4 and RPA increased signal compared to *yku80* deletion relative to their respective WT controls. That may be explained by Ku blocking Exo1, while Rad9 and Nej1 inhibit Sgs1/Dna2 (e.g. Mojumbar *et al.* Cell Rep 2019).

With the inclusion of Fig. 1B, I find myself asking throughout the paper, how do these Rad9 mutants behave in vivo with ku80Δ and xrs2Δ? I don't know it the inclusion of this figure adds. Is BSA the -ctrl , and equivalent to rad9Δ ?

Yes, BSA is used as negative control in these *in vitro* acetyltransferase assays and represents the -Rad9 control (by extension corresponding to *in vivo rad9Δ*). We have repeated the assay to more clearly define the lesser effect of Rad9 Tudor and BRCT point mutants (**new Fig. 1B**). As mentioned before, we feel this is a nice introduction to the topic of the study, linking it to previous concepts in the literature. On the other hand, we feel that incorporating these Rad9 point mutants in the subsequent *in vivo* experiments would not add much as it is known that they are critical for Rad9 function (e.g. Nnakwe et al. DNA Repair 2009).

Fig. 3E and F could be put in Supp and mentioned more in the Discussion. – They break the flow of the xrs2Δ rad9Δ ku80Δ story.

We understand the suggestion but since Reviewer 3 below had an opposite suggestion, to move related supplemental material from S3D-E Fig to the main figure, we could not see how to address both suggestions. Thus, we left the panels as originally split between main and supplemental figures.

Fig. 5C and D, it is difficult to tell if pNEJ1-3KR is the same as WT or not included – change of color for one of them would be clearer.

We understand the visual problem as the lines are overlapping. We have now changed the weight of the lines in all the graphs presented in the manuscript for visual clarity.

Reviewer #3:

Ahmad et al. present an interesting study addressing the functions of the histone acetyltransferase NuA4 at DNA double strand breaks (DSBs), a relatively less studied aspect of DSB repair regulation. It builds from the group's prior findings that suggest a competition between Rad9 (53BP1) and NuA4 (Tip60) at DSBs, which sets up the idea the NuA4 promotes resection, including that it is recruited by virtue of its Tel1-like subunit Tra1 in yeast interactions with Xrs2. Here they extend their findings by examining yeast G1 cells and interactions with the predominant G1 DSB repair pathway, nonhomologous end joining (NHEJ).

Results are novel and derived from experiments that are mostly well performed and yields new insights that Rad9 as well as Ku inhibit NuA4 binding in G1. Evidence is presented that both Lcd1/Ddc2 and Xrs2 collaborate to recruit NuA4 to DSBs. NuA4 activity is at least partially responsible for the resulting increased resection in G1, although definitive studies are impaired by the essential nature of NuA4 for cell viability. Novel acetylation targets for NuA4 are nominated as the NHEJ proteins Nej1 and Yku80. These data support a model in which NuA4 and NHEJ proteins, not unlike Rad9 and NuA4, have an antagonistic relationship in regulating DSB repair.

Overall, this is a quality study with interesting and novel findings that recommend it for publication. However, some significant issues should be addressed prior to publication.

We are of course delighted that the reviewer finds our study interesting, well performed with new quality insights, and thinks that our novel findings support the model of an antagonistic relationship between NuA4 and NHEJ in regulating DSB repair. The reviewer recommends publication after some revisions.

1) In Fig. 2A, I assume the authors did not show rad9 mutant alone because it has already been shown previously? I think it is important and relevant to be able to see the quartet of 4 relevant mutants (WT, two singles, and one double) all on one plot. Similar comments apply to other plots where the reader is asked to compare traces between figures to get a full understanding of the strain relationships, which can be difficult to do accurately as presented.

We understand the reviewer's comment about comparing mutants in different plots. We have now made an effort to repeat experiments with larger sets of mutants in single plots when possible, to give a clearer picture. This is now included as **new Fig. 2C-D, 3C-D, 4A-B**.

2) Fig. 3 is a weaker part of the manuscript and difficult to interpret. The impact of the pMRE11-NLS plasmid clearly has a strong impact, but like my other comments I am having difficulty understanding if it provides a complete suppression of the xrs2 mutant phenotype regarding NuA4 recruitment. As above, this is because some allele combinations do not seem to be reported or are not on the same plot. Specifically, I am interested if xrs2 mutation still has any effect at all in strain carrying pMRE11-NLS? These results are important for the final

interpretation that _both_ Xrs2 and Lcd1/Ddc2 independently recruit NuA4.

This is a good point also raised by reviewer 2. As discussed above and suggested by Reviewer 2, we have now included a new experiment with pMRE11-NLS-X85 that completely bypasses Xrs2 function for Mre11 binding to the DSB and resection (**new Fig. 3C-D, S3B Fig**). These data confirm in a more complete way the recruitment of NuA4 without possible interaction with Xrs2. In the situation of Xrs2 bypass, NuA4 recruitment is increased by resection and requires Lcd1/Ddc2 (**new Fig. 4A-B** vs C-D).

3) Continuing with Fig. 3, the strains in Fig. 3E,F having an accumulation of 5 different genetic alterations making it very difficult to understand and to be confident what the key interpretations are (even with the supporting text). Moreover, the key experimental manipulation is again missing, specifically LCD1 WT vs. lcd1 mutant with all of the other alleles in Fig. 3E,F still present. The authors seem to wish us to compare to other LCD1 WT strains, but those are not in the same complicated background. Only a side-by-side comparison with and without LCD1 can show its specific effect.

As discussed above for reviewer 1, we have shown in a previous report that *LCD1/DDC2* deletion by itself had no apparent effect on NuA4 recruitment in asynchronous cells (PNAS 2018). We have now included new more complete data in **Fig. 4A-B/S4C Fig** to make it clearer to conclude that recruitment of NuA4 in conditions of Xrs2 bypass and increased DNA end resection is dependent on Lcd1/Ddc2 (compare **new Fig. 4A-B** with C-D)

4) The main paper has only 6 Figures and one is a model only, which makes me wonder if some of the results in Supplemental ought to be promoted to the main paper. Fig. S3E may help address some of my concerns related to Fig. 3 above, in having more allele combinations, although I am still not certain all of the precise +/- Lcd1 and +/- Xrs2 combinations can be found.

As this is opposite to reviewer 2's suggestion to move some panels of Fig. 3 to supplemental, we decided to leave the initial distribution. Furthermore, we feel that addition of the **new more complete dataset in Fig. 4A-B** merged with old 3E-F panels (now 4C-D) makes the comparison of Xrs2, resection and Lcd1/Ddc2 effects on NuA4 recruitment clearer.

5) If Fig 4., I would be interested to know if a specific SSA joint could be detected as forming within arrested G1 cells; since Rad52 is recruited, can productive repair be completed in G1 by a non-NHEJ mechanism? This is not a major point of concern, however.

We really thank the reviewer for this great suggestion as we have now included new important results that supports such a claim (**new fig. 5F**). We used the yeast strain tNS-2333 that has 2 homologous segment 20kb apart on the same chromosome with an HO cut site in between (Jain *et al.* PLoS Genet 2016) and introduced an *esa1-ts* allele in it. We also deleted *RAD51* to block possible gene conversion events and compared HO cut survival by spot assays with Rad52 which is essential for SSA. Strikingly we found that the *esa1/rad51* strain is as sensitive to the HO cut as a *rad52* mutant strain. This sis strongly suggesting that NuA4 favors the resection-dependent SSA repair pathway that could occur in G1 when the NHEJ machinery is disrupted.

6) Unless I missed finding it in the provided review materials, it is difficult or impossible to examine the evidence supporting the claim that the 3 lysine residues in Nej1 are in fact acetylated and to what extent in yeast cells. There is simply a table of identified sites in Supplemental. The mutant data are interesting but paint a picture of a pleiotropic effect that is not entirely attributable to acetylation (e.g. the impact of the K to R mutations on Lif1 binding), so it is important to be able to judge the quality of the evidence for acetylation independently of mutant effects that might be mediated by folding or other mechanisms. Might the anti-acetyl-lysine antibody be of use here, e.g. applied to WT and mutant Nej1 as was done for Ku?

We understand the comment and made efforts to provide more information. We have now included 2 new experiments as **new Figure 6A-B.** First, we show by radioactive acetyltransferase assay on gel that the purified NuA4 complex clearly acetylates the Nej1 protein *in vitro* **(6A).** Then, we use immunoprecipitation with an anti-acetyl-lysine antibody from whole cell extracts that Nej1 is acetylated *in vivo* and the level does not seem to significantly change after DNA damage with MMS **(6B).** Finally, we have now included in the **new S2 Table** the full list of acetylated peptides confidently detected by mass spectrometry of Nej1 purified from WCE of WT and *esa1* cells, after trypsin digestion. Overall, the total peptide sequence coverage obtained by mass spectrometry was 83 and 77%, respectively. These results were obtained by over-expression of a GST-Nej1 fusion in yeast cells. Unfortunately, expression of WT and mutant Nej1 from the low copy plasmids used in the subsequent experiments (more physiological levels) does not seem enough to detect the protein after anti-Kac IP or ChIP-qPCR experiments (see above).

7) No description or illustration is presented of where in the Nej1 protein the putative acetylated residues lie with regard to functional domains or three-dimensional structure. Such information would be a helpful adjunct to understanding the possible mechanistic consequence of any acetylation of those residues.

We thank the reviewer for this helpful suggestion. We have now included a cartoon of Nej1 depicting its known functional domains and the locations of the mapped Kac sites (**new Fig. 6C**).

8) The authors use Rfa1 ChIP throughout as a surrogate for resection. This is reasonable for a study that uses ChIP extensively, but in one or two key conditions/comparison I would welcome DNA-based resection experiments in cells held in G1. Fig. 3E does something along these lines but the results are not compelling and unless I missed it the method used there is never described.

We are sorry for the missing details in the method section. We have now included the information. We have also included new experiment directly measuring DNA end resection (as in original Fig. 3E) in G1 blocked cells carrying the *rad9, yku80* and/or *xrs2* mutant alleles (**new S2D Fig to compare Rfa1 signal in new Fig. 2D, see also direct DNA measurement in new Fig. 4A**)

9) Throughout, the figures with line diagrams of signal at distances from the DSB use thin lines and tiny points that can be difficult to discern. At a minimum, thicker lines would make colors

easier to discern, but I also recommend the use of visible data points with symbols that allow another way of telling traces apart.

As mentioned above for reviewer 2 we have now made the lines ticker in all graphs to improve visual clarity. We feel that adding visual data points with symbols would make the graphs too busy when multiple strains are compared.

10) The systematic gene names for the yeast Ku subunits have been YKU70 and YKU80 for many years now; I do not recommend even including the names HDF1 and HDF1 at this point.

Okay, we removed those names, and added *DDC2* to *LCD1* as suggested by reviewer 1.

11) Stylistically, I strongly recommend that the authors use proper scientific convention in always referring to results of the current study in past tense (i.e, "Deletion of XYZ1 resulted in", not "results in", "The effect was similar when", not "is", etc.).

We went through the text to make it more uniform using the past tense.

12) There are grammatical errors throughout the paper, some of which I list below (there are likely more).

Page 5, .line 86, "antagonize", not "antagonizes".

Page 5, line 92, "allowing", not "along".

Page 5, line 96, "disrupts", not "disrupt"

Page 8, line 157, "measured _by_ Rfa1/RPA".

Page 8, line 162, "slow down _the_ rate"

Page 11, line 221, "terminus", not "terminal"

Page 16, line 321, "thereby", not "there by"

Sorry for these numerous mistakes/oversights, we asked a native English speaking person to help and we were more careful at verifying the text. Corrections were made.

Have all data underlying the figures and results presented in the manuscript been provided?
Large-scale datasets should be made available via a public repository as described in the PLOS Genetics [data availability policy](), and numerical data that underlies graphs or summary statistics should be provided in spreadsheet form as supporting information.
Reviewer #1: ***No:*** *ChIP data and other graphed data is only presented as an average +/- error on graphs. The underlying data is not in presented in numerical form, either averaged or individual experiments.*

We are not sure what would be needed here. The graphs and error bars do present the values of each data points (error bars are range between replicates). Excel files with numerical values could be provided but we feel that would be redundant and clutter the supplemental information since there are so many qPCR datasets.

Reviewer #2: Yes

Reviewer #3: ***No:*** *I was unable to find any detailed data supporting the discovery of Nej1 acetylated residues by mass spec, just a summary table of identified sites. See my review for additional comments; I find it ~impossible to judge the evidence that the sites are truly acetylated.*

See response to Reviewer 3's comment above.
