# Supplementary material for: Antagonistic relationship of NuA4 with the non-homologous end-joining machinery at DNA damage sites
Source: PLoS Genet. 2021 Sep 20;17(9):e1009816. doi: 10.1371/journal.pgen.1009816 (PMC8483352; doi:10.1371/journal.pgen.1009816)
Supplement: Attachment — Submitted filename: Response to reviewers-2.pdf [file pgen.1009816.s011.pdf]

**Response to reviewers' comments:**

**<u>Reviewer #1:</u>** *The paper has improved, but I still think that some conclusions are overstated and should be modulated.*
*(1) Concluding that NuA4 has a G1 role. The ChIP data was all collected in an un-natural condition, where G1 end protection is abolished and thus excessive resection is occurring, is misleading. For example, the conclusion at the end of the abstract "these results…suggests a role of NuA4 in alternative repair mechanism that involves DNA-end resection in G1". The NuA4 role in G1 is only shown in the context of a mutant situation with increased DSB resection. The authors should stick to the conclusion that NuA4 acetylation antagonizes NHEJ through direct acetylation of NHEJ factors (Nej1 shown here). And that in cases of where DSB ends are available, be that in G1 due to a mutant situation, or maybe stochastically at some ends, or S/G2, NuA4 is recruited via interaction with Ddc2 and facilitates resection.*

This point of the reviewer is partly answered by reviewer 3's counter-argument below (underlined). Things are complicated at DSBs as so many factors are present to orchestrate the response. Our point is that NuA4 can be recruited during the G1 phase and can assist repair in other pathways than classical HR (SSA). Thus, its recruitment normally occurring in S/G2 is not due to a cell cycle-regulation by direct modification of/signaling to NuA4. So in specific circumstances, it can play a role in DSB repair in G1. Nevertheless, we made changes in the text to acknowledge that the conditions in which we see recruitment and function in G1 are in a mutant background leading to high resection which does not normally occur.

*(2) Throughout the manuscript, the authors conclude that Xrs2 is required for NuA4 recruitment. Though recruitment is technically "dependent on Xrs2", The data show that the Xrs2 results are mainly due to its role in Mre11 import and Mre11-dependent resection. It would be more informative to state that NuA4 recruitment is "dependent on MRX-mediated resection" or "dependent on Xrs2's role in enabling MRX-mediated resection". Key lines where this more accurate wording should be used are in the abstract line 31 and intro line 93.*

We understand the reviewer's comment but would like to point out that we demonstrated a direct DNA damage-induced phospho-dependent physical interaction between Xrs2 and the NuA4 complex in our previous work (Cheng et al. PNAS 2018). Since NuA4 was not recruited at DSBs in the absence of Xrs2 it was quite logical to propose that the physical interaction is responsible for NuA4 recruitment. In the present study, the data suggest that Lcd1/Ddc2 could also lead to NuA4 recruitment in the absence of Xrs2, as suggested by previous data showing an in vitro physical interaction between NuA4 subunit Tra1 and Lcd1/Ddc2 (Cheng et al. PNAS 2018). Nevertheless, we modified the wording in the two lines indicated by the reviewer.

*Other writing issues:*
*Rad9 should not be characterized as a NHEJ factor (abstract and other places). But the point that Rad9 binding to resected DNA can directly inhibit NuA4 acetylation is interesting and can be made.*

We understand the reviewer's argument and changed the text at several places to avoid naming Rad9 as a NHEJ factor per se.

**<u>Reviewer #2</u>**: *The authors have performed additional experiments and responded to reviewer's comments appropriately. They have revised manuscript and explain their work and model more clearly.*
*The experiments with pMRE11-NLS-X85 are a nice addition.*

We thank the reviewer for appreciating our revised version and added the very nice experiment/data that he/she suggested.

**<u>Reviewer #3</u>**: *Ahmad et al present a revised version of their manuscript exploring the recruitment and activation of the NuA4 acetylase at DNB double strand breaks in G1, with a major conclusion that NuA4 and NHEJ promoting factors show an antagonistic relationship in which NuA4 acts to promote resection and is held in check by Rad9 and NHEJ proteins. In turn, NuA4 acetylates NHEJ proteins.*

*I was previously positive about the manuscript but had noted several problems with the presentation and some of the data. These have been diligently and consistently addressed by the authors so that I now see the manuscript as suitable for publication in PLoS Genetics. Most of these related to clarity of presentation and data support, but the revised manuscript also adds interesting new data on an SSA model and enhanced acetylation studies. The study still relies at points on complicated interpretations of multiply mutated strains, but the analysis is thoughtful and meticulous such that I think the conclusions are solid, in additional to being novel and interesting to those studying DSB repair.*

We are delighted that the reviewer appreciated our modifications and new data and that he found our work meticulous, solid and novel/interesting.

*With respect to other reviewers' comments, <u>Reviewer 1 was most negative. I agree that in some ways the study is weakened by a particular focus on G1 where the exact role of some the protein functions might be questioned, e.g., if they are only evident when other proteins are mutated, are they important? However, I tend toward the view that the interactions are reproducible and likely part of the complicated orchestration of protein activities at DSBs in general. As such, perhaps unlike Reviewer 1, I found the overall collection of findings to be coherent and cohesive as drawn in the final model.</u>*

We certainly welcome the reviewer's comment that counterbalanced reviewer's 1 position/comments. We agree that the processes at play are likely part of a complicated network of activities at DSBs. We are delighted that he/she finds our findings coherent and cohesive.
