# Supplementary material for: Antagonistic relationship of NuA4 with the non-homologous end-joining machinery at DNA damage sites
Source: PLoS Genet. 2021 Sep 20;17(9):e1009816. doi: 10.1371/journal.pgen.1009816 (PMC8483352; doi:10.1371/journal.pgen.1009816)
Supplement: Attachment — Submitted filename: Ahmad et al review .docx [file pgen.1009816.s009.docx]

Ahmad et al., describes the antagonistic relationship between the Non-Homologous End-Joining machinery at an HO-induced DSB. The authors also identify Ku and Nej1 as NuA4 targets and through mutagenesis they show data supporting the model that acetylation helps remove NHEJ factors as a mechanism to promote HR. The experiments are high quality and the work contributes to the DSB field. A few controls are requested and further explanation is needed in some areas.

- The interpretation of data with *xrs2*Δ needs revision as the MRX complex does not assemble at breaks and other factors binding to MRX would not be recruited either. Line 160 (p.8) ‘… due to the loss of interaction with Xrs2, or the loss of resection itself. ‘ is a generalization and does not capture the complexity as *mre11*Δ would give the same result. Comparing *xrs2*Δ to *mre11*-H125N indicates that the nuclease activity is not required for NuA4 recruitment. As the authors show NuA4 interacts with Xrs2-N-term (PNAS) –incorporation of pMRE11-NLS-X85 or X164 into their work would clarify a number of questions.

Mre11 is not recovered at the DSB in *xrs2*Δ and it is largely reduced in *pMRE11-NLS*, but restored when fragments of Xrs2, X85 or X164, are fused to Mre11. Presumably these would not interact with NuA4. For Fig. 3, include pMRE11-NLS-X85 or X164. This would clarify the interpretation of data in Fig. 2 and 3.

For Fig. 3A, does the IP WT + pRS416 (EV) in G1 give the same enrichment as WT in Fig. 2? Please include this as well *xrs2*Δ *rad9*Δ *ku80*Δ +pRS416 (EV). This will help with the interpretation of data between Fig. 3A and Fig.2C.

Does *xrs2*Δ have the same additive effect with *rad9*Δ that y*ku80*Δ has for NuA4 Rfa1 and Rad52 localization (Fig. 1G-H and 4B)?

With the inclusion of Fig. 1B, I find myself asking throughout the paper, how do these Rad9 mutants behave in vivo with *ku80*Δ and *xrs2*Δ? I don’t know it the inclusion of this figure adds. Is BSA the -ctrl , and equivalent to *rad9*Δ ?

Fig. 3E and F could be put in Supp and mentioned more in the Discussion. – They break the flow of the *xrs2*Δ *rad9*Δ *ku80*Δ story.

Fig. 5C and D, it is difficult to tell if pNEJ1-3KR is the same as WT or not included – change of color for one of them would be clearer.
